# Rheological Performance and Differences between Laboratory-Aged and RAP Bitumen

**DOI:** 10.3390/ma17163954

**Published:** 2024-08-09

**Authors:** Noemi Baldino, Olga Mileti, Ylenia Maria Marchesano, Francesca R. Lupi, Domenico Gabriele, Massimo Paolini

**Affiliations:** 1 Department of Information, Modeling, Electronics and System Engineering (D.I.M.E.S.), University of Calabria, Via P. Bucci, Cubo 39C, I-87036 Rende, CS, Italy; o.mileti@dimes.unical.it (O.M.); marchesanoy@gmail.com (Y.M.M.); francesca.lupi@unical.it (F.R.L.); d.gabriele@unical.it (D.G.); 2 Valli Zabban SpA, Via di Le Prata 103, 50041 Calenzano, FI, Italy; paolini@vallizabban.it

**Keywords:** PAV, soybean oil, viscosity, oxidation, aging, pavement

## Abstract

Traditional recycled asphalt pavement (RAP) binder extraction is not a cost-effective and sustainable option for a quick field study because it requires the use of a huge amount of solvent. Hence, most of the studies on asphalt pavement are carried out with laboratory-aged bitumen in accordance with well-established procedures, i.e., the pressure aging vessel (PAV). Unfortunately, some studies highlight the differences between bitumen aged in the laboratory and in service because it is difficult to reproduce extreme conditions such as real conditions, both atmospheric and load; and this also affects the choice and use of rejuvenators, sometimes compromising the interpretation of results. This study aims to compare the thermo-rheological behavior of a 70/100 bitumen aged with the PAV and two different binders extracted by RAPs. The rheological performances of bitumens were compared in temperature and by dynamic oscillatory tests and steady-state tests, resulting in strength and viscosity values higher for samples with RAP binders compared to the PAV sample. The same bitumens were tested with the addition of a 3% *w*/*w* of soybean oil (SO). The results show a decrease in the moduli and viscosity at all the temperatures investigated when SO is added to the laboratory-aged bitumen, while no appreciable differences are evident on naturally aged samples added with SO. Differences were evaluated in terms of cross-over frequency and rheological parameters. Furthermore, the SO effect showed substantial differences, especially in viscosity values, indicating that the study of regenerated or modified bitumen from aged bitumen still requires study, as current standard techniques and procedures cannot emulate real aging conditions well.

## 1. Introduction

The use of road pavements leads over time to various road surface defects, all of which are related to the aging of asphalt pavements [1]. The study of the aging of asphalt pavements is a complex topic to investigate due to the many environmental factors involved [2]. There are mainly two distinct phases of the aging phenomena: short-term and long-term aging. Short-term aging occurs in the asphalt production and transport phases, and long-term aging refers to what happens to bitumen during its service life [3,4]. Asphalt is a complex material, and environmental factors, such as oxygen, moisture, sunlight and ultraviolet radiation, lead to physico-chemical reactions such as volatilization, oxidation and polymerization, resulting in a reduction in light components such as aromatic, saturated and asphaltene, and an increase in heavy asphaltene components. Macroscopically, this leads to increased asphalt viscosity, increased brittleness, decreased toughness, etc., [2] and, more generally, to a change in the rheological properties of asphalt binders [2,5]. Understanding and studying the mechanisms associated with oxidation phenomena due to the aging of bitumen is crucial to understanding the origin of changes in the rheological properties of aged bitumens [6]. Rheological indices, such as penetration and softening point measures, are used to quantify the asphalt performance or to evaluate the effect of aging.

The mechanisms classically observed during bitumen aging are physical and steric hardening (reversible mechanisms), loss of low-weight (volatile) components through evaporation, and oxidation, resulting in changes at the molecular level that cause a change in SARA fractions, in particular, with a decrease in aromatics and an increase in asphaltenes [3]. The final effect is a decrease in penetration degree and an increase in viscosity, mainly related to the formation of polar groups [3,4,7]. Under the same aging conditions, different bitumens may age differently due to different compositions [4]. Furthermore, the aging effect can be slowed down by the humidity of the environment, with more humid environments leading to slower aging [4]. Although the literature says that the more superficial layers tend to deteriorate faster than the layers below, we recommend checking that the aging of the area on top and the area below the bituminous mixture does not show differences [4,8].

To understand how recycled asphalt pavement (RAP) binder behaves over time and how it can be recycled, researchers have developed several methods for simulating its aging: pressure aging vessel (PAV) combined with the rotational thin film oven test (RTFOT) method to evaluate long and short-term aging, respectively, as well as compacted mix aging (CMA) and loose mix aging (LMA). The literature suggests that LMA is a better technique than CMA to simulate asphalt pavement aging [9]. RAP binder (RAPb) simulated using the different above-mentioned techniques can vary in characteristics, and this can in turn influence the performance of the base binder [9]. This variation can affect the parameters used for blending and recycling in asphalt pavements, including the amount of RAPb that can be added to the base binder and the performance of these mixtures in terms of rupture and fatigue [10].

During the PAV aging, all asphalts show a rapid initial reactivity followed by a slower phase at a constant rate [11]. It has been shown that the chemical reactions of hydrocarbons characterising the fast and slow phases of aging reactions are fundamentally different [11]. Despite the aging study using the PAV technique, the results obtained do not agree with what happens naturally during the natural aging process of the binder [12]. In particular, during natural aging, alcohol functional groups are formed that are responsible for the change in viscosity of the bitumen. The formation of sulphoxides as a result of oxidative aging is associated with increased viscosity [11]. This phenomenon is not detected during laboratory aging, and it was found that the combined use of the RTFO/PAV technique is only equivalent to an 8-year binder aging at 75 mm depth from the surface [5,11,12]. Also, in their study on the aging of bitumen, observe how the PAV and RTFO techniques used to study the aging of asphalts result in different rheological behaviors to that found with natural aging [13].

Currently, the rheological properties of asphalt aged are mainly analyzed by complex shear modulus, G*, and phase angle, δ, and it was found that bitumens from different sources and/or with different grades of penetration age according to different characteristic properties [2]. Particularly, this is a result of the different amounts of asphaltenes originally present in origin, which are transformed differently during the aging phase [2]. With the aim to recycle asphalt pavement and improve the performance of high-RAP mixtures, it is fundamental to understand the behavior of aged bitumen and to employ a rejuvenator, due to the high crack susceptibility of RAP [14,15]. The use of rejuvenators is also essential when a high amount of RAP is employed. In particular, the rejuvenator addition is used to restore the condition of RAP to give it mechanical and chemical properties close to its initial ones [16]. Two important mechanical properties are bitumen fluidity and softness, which ensure workability and spreadability [17]. The use of vegetable oils represents an interesting area of research in the restoration of aged bitumen to replace petrochemical-based additives and then improve environmental impact [15]. Vegetable oils are characterized by a certain quantity of triglyceride, which consists of unsaturated fatty acids. These triglycerides are similar to the light components of asphalt binders, giving them good compatibility [15]. Moreover, it was found that triglycerides could also activate other modifiers that subsequently enhance the performance and compatibility of modified asphalt [15]. 

Vegetable oils can be incorporated into composite asphalt modifications alongside other additives. They function as a surface activator, enhancing the compatibility of other asphalt additives with the binder. Vegetable oils can mitigate aging, accelerated by ultraviolet radiation and heat, which stiffen and embrittle asphalt binders, compromising their resistance to fatigue and thermal cracking. The action of the vegetable oil is caused by providing lighter components that interact physically with the aged binder [15].

A rejuvenator is necessary to use a greater RAP percentage in road construction, but the study of this modification is generally conducted using laboratory-aged bitumen. The variability in fatty acid composition among vegetable oils significantly influences their physical and chemical properties. Consequently, different oils can interact with bitumen in distinct ways [15].

In particular, there are literature studies that report the use of soybean oil as rejuvenating and as an agent capable of restoring the characteristics of RAP [14,18]. In addition, it is a low-cost vegetable oil. The addition of soybean oil led to a decrease in the consistency of RAP, confirming the fluxing effect [17]. When compared with other fluxes, smaller quantities are sufficient to achieve optimal rheological properties [17].

Therefore, in this work, both the effects of aging and the effects of SO addition were studied to understand how laboratory aging simulates the rheological properties of RAP binders and how vegetable oil is able to restore the binder conditions. Two binders from two distinct RAP sources were investigated and compared with a PAV-aged bitumen to address this. The RAP binders were named as soft and hard based on their origin: one from virgin aggregates and the other from recycled aggregates. All the results were evaluated by rheological parameters like the complex modulus (G*) and phase angle (δ) obtained through oscillatory analysis at high temperatures and steady-state conditions. Additionally, the binders were modified with SO at 3% *w*/*w*, and their altered thermo-rheological properties were analyzed.

## 2. Materials and Methods

### 2.1. Materials

The analyzed samples are a virgin bitumen API 70/100 (identified in the text as API), the virgin aged by PAV (identified in the text as PAV), and two kinds of reclaimed asphalt pavement binders (RAPbs), RAP_H and RAP_S, according to their origin. The pavements of both RAPbs were obtained with bitumen 70/100. RAP_S and RAP_H were obtained by two different road pavements: the first was constructed with virgin aggregates and the second with RAP aggregates [1]. The asphaltene content of aged bitumen was evaluated by quantitative gravimetric analysis without calcification and was of 39.08 ± 1.20%, 27.06 ± 2.24% and 32.27 ± 2.01% for RAP_H, RAP_S and PAV, respectively [19].

The SARA analysis of virgin bitumen is reported in Table 1.

PAV aging procedure was conducted at 2.1 MPa, 100 °C for 20 h, according to ASTM D6521 and the literature [2,9]. All the aged bitumens were modified with new SO kindly supplied by Marchemical Lab. S.r.l. (Castrovillari, Italy).

The samples were prepared following a few simple steps. First, 100 g of bitumen was heated up to 180 °C on a hotplate stirrer (IKA C-MAG HS 7, IKA-Werke GmbH & CO. KG, Staufen, Germany) with a thermocouple (IKA ETS-D5, IKA-Werke GmbH & CO. KG, Staufen, Germany) under stirring through a mechanical paddle stirrer (Heidolph RGL 100, Heidolph Instruments GmbH & Co. KG, Schwabach, Germany). Only for the modified samples, SO was added to the heated bitumen at a 3% weight ratio under constant stirring. The mixture was maintained under agitation for 20 min [20]. All the samples were then cast into circular silicone molds, with the diameter of the rheometer plate for the analysis according to [21].

### 2.2. Experimental Methods

All bitumen samples were characterized by a rheological approach with a Dynamic Shear Rheometer, DSR 500 (Rheometric Scientific, Piscataway, NJ, USA), equipped with a Peltier system (±0.1 °C) for temperature control. All measurements were performed with a parallel plate geometry (gap = 2 mm, Φ = 25 mm) according to [21]. Frequency sweep tests, in linearity, were performed at 25°, 40°, 60° and 80 °C, in a frequency range between 0.1 and 10 Hz. Temperature ramp tests were carried out at 1 Hz in the temperature range of 20–100 °C, with a ramp of 1 °C/min. Flow curves were carried out at 60° and 75 °C, increasing the stress from 1 Pa up to 3000 Pa in steady-state conditions.

All samples were studied at high temperatures performing temperature ramp tests at 1 Hz, heating the sample at 1 °C/min from 25 °C up to 100 °C, in linear conditions previously evaluated by stress sweep tests [22].

Rheological data of time–temperature superposition (TTS) were interpreted by the Winter gel model [23]:

(1) $$G^{*}=k\cdot \omega^{n},$$

where G* is the complex modulus, obtained as $\sqrt{G^{\prime 2}+G^{\prime \prime 2}}$, *k* is a measure of the gel strength, and *n* is a measure of the structuration degree. In particular, a low value of *n* corresponds to a high degree of structuring.

## 3. Results and Discussion

### 3.1. Rheological Results

The viscoelastic properties of bitumen, both laboratory-aged and naturally aged, were analyzed. Researchers have proposed the TTS method as a tool to describe the linear viscoelastic behavior of bitumen across a wider range of frequencies. The core principle of TTS relies on the assumption that the relaxation mechanisms remain constant regardless of temperature, with only the relaxation times changing. By applying TTS, the behavior observed at shorter times (high frequencies) can be aligned with the behavior observed at lower temperatures, and, conversely, the behavior at longer times (low frequencies) can be aligned with the behavior at higher temperatures [21,24]. This technique allows us to generate “master curves” for G′ and G″ for all the bitumens analyzed using a horizontal shift factor (αT). In this way, the TTS principle provides a valuable tool for understanding the behavior of bitumen across a wide range of frequencies. A reference temperature of 40 °C was chosen, as it represents a typical average surface temperature for regions in the southern Mediterranean area [21]. 

Furthermore, the crossover frequencies (f_c_) for all samples were obtained from the TTS data, i.e., the frequency at which there is an inversion of the moduli and, therefore, the predominance of G′ compared to G″. The f_c_ is evaluated when 45° of the phase angle is reached.

**Figure 1:**
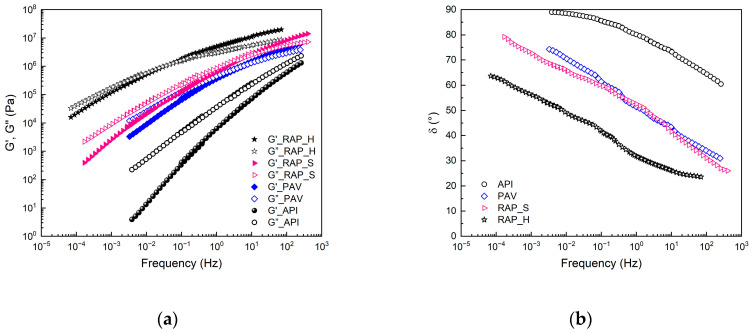
TTS diagram: (**a**) dynamic moduli (G′ and G″); (**b**) phase angle.

The TTS diagrams are reported in Figure 1, in terms of the storage modulus, G′, and loss modulus, G″, (a) and the phase angle (b). With the TTS method, the behavior at high frequencies obtained from the TTS principle can correspond to the behavior of low temperatures, whilst the behavior at low frequencies can correspond to the behavior of high temperatures [25,26]. 

It can be seen from Figure 1a that the API bitumen exhibits liquid-like characteristics over the entire frequency range investigated. Liquid-like behavior is characterized by a loss modulus (G″) greater than the storage modulus (G′), indicating a predominant liquid contribution, and a phase angle (δ) exceeding 45 degrees [21,27]. In particular, looking at the slope of G′ and G″, it can be observed that it is dependent on higher frequencies. As a result of the aging procedure, the characteristic modulus of the bitumen shifts to higher values and, observing well, the slope of the moduli is lower, indicating a more structured behavior, according to the increased asphaltene value. 

In particular, observing the parameters of the Winter gel model reported in Table 2, it can be seen that the gel strength increases one order of magnitude for the sample aged in the laboratory, but its *k* value is well below the value obtained for both RAPbs, indicating a value of hardening that is higher for the naturally aged binders compared to PAV, even if there is a similar asphaltene content for the API and RAP_S samples. The difference in the gel strength can be related to the light component in which the asphaltenes are dispersed. As concerns the *n* value, it is possible to note that it sharply decreases with aging when comparing the API and PAV samples. The *n* value shown by RAP_S is similar to PAV, suggesting a similar frequency dependency and structuring degree. In particular, it is possible to note that PAV and RAP_S have similar phase angles (Figure 1b), pointing to a similar structure as confirmed by the *n* values. This trend is in agreement with the percentage of asphaltenes in PAV and RAP_S and with the Winter model because the *n* value is related to the number of interacting structures rather than to the asphaltenes. In fact, it is well known that the structuring of the bitumen is strongly related to the number of asphaltenes within the bitumen. With low asphaltenes, the *n*-value is high and, therefore, less structured, with high asphaltenes, the *n*-value is low and more structured.

The *n* value for RAP_H is different, which is lower compared to the other two aged samples due to the high amount of asphaltenes, indicating a strong frequency independence accompanied by a low ability to relax the stresses observable in Figure 1b. The value found and the low dependence of the moduli on the frequency confirm the predisposition of the material to give rise to cracking phenomena.

Furthermore, by observing the G′ and G″ trend with the frequency, it is possible to note that no crossover of the moduli is present for the API, while a crossover is observed in the aged bitumens beyond which the behavior of them becomes solid-like and more susceptible to stresses. Comparing the PAV sample with the rheological behavior of the RAPs studied, a similar frequency crossover for RAP_S and PAV was found, while the RAP_H shows a crossover at a very low frequency, as reported in Table 2. 

It is evident from the data obtained from the frequency tests that laboratory aging is only partially capable of emulating natural aging. In fact, it increases the asphaltenes, but the hardness of the bitumen cannot be emulated, at least with the classic procedure, as suggested by the value of *k* obtained, an order of magnitude lower than that obtained in the laboratory. This behavior aligns with findings in the literature using similar laboratory techniques to PAV [12].

The viscoelastic properties of the samples were also studied by using thermal ramps at 1 Hz, and, from the data, the T_c_ was obtained. The T_c_, reported for all samples in Table 2, indicates the passage of the bitumen from the gel to the sol state, and vice versa, and is obtained at the temperature value at which G′ is equal to G″, therefore, when the phase angle reaches 45°. Observing the moduli trend with temperature for the API sample, a liquid-like behavior throughout the investigated temperature range is evident, and, only after aging (PAV sample), an increase in the dynamic moduli is noted with a crossover at 33.5 ± 1.0 °C.

**Figure 2:**
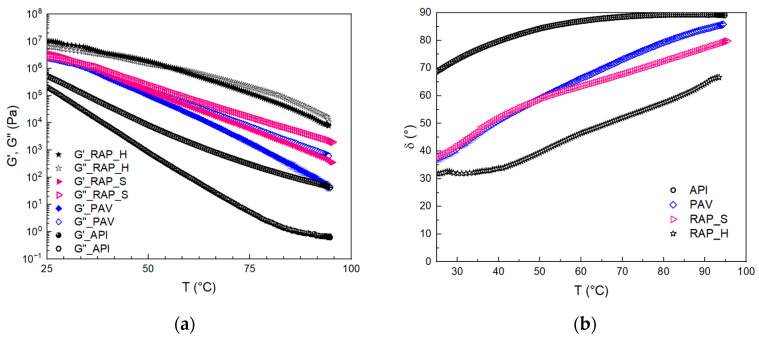
Time Cure diagram: (**a**) dynamic moduli (G′ and G″); (**b**) phase angle.

The two samples RAP_H and RAP_S have different behaviors compared to the PAV binder. Particularly speaking, RAP_S shows similar behavior to the PAV only at low temperatures even if has higher moduli and has a crossover from gel to sol behavior at a temperature close to the PAV, as it is possible to note from the phase angle (Figure 2b) and the values reported in Table 2. At approximately 50 °C, RAP_S exhibits higher moduli than the PAV, demonstrating a less temperature-sensitive structure. This is corroborated by a lower δ value, indicating reduced rutting susceptibility as also reported by [14]. This behavior deviates from PAV trends and highlights the distinct characteristics of naturally aged bitumen. The RAP_H moduli are the highest at all the temperatures, and the transition happens at higher temperatures compared to the other two samples, indicating a strong hardness and poor susceptibility to temperature. The trend with temperature is corroborated by the measurements of viscosity that can point out the ability of aged bitumens to be laid down. 

**Figure 3:**
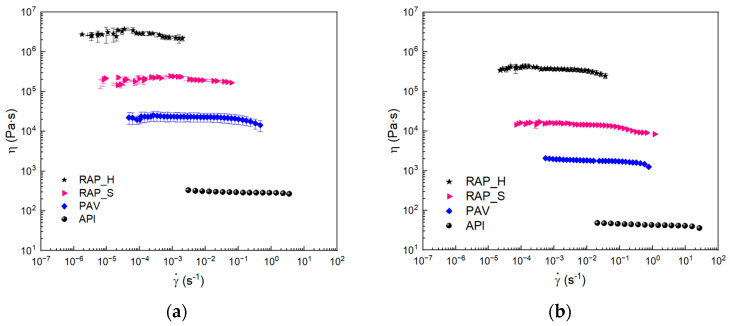
Flow curve plot: (**a**) at 60 °C; (**b**) at 75 °C.

The viscosity measurements were performed at 60 °C and 75 °C, and the results are reported in Figure 3a,b. It can be seen that, at both temperatures, PAV aging leads to an increase in the viscosity of the bitumen, although it does not change its behavior, which remains almost Newtonian over the range of shear rates studied. Again, the RAP_H and RAP_S samples are at least an order of magnitude more viscous than the PAV-aged one, indicating that the real aging phenomena occurring are more important than those obtained from laboratory aging, according to the literature [28]. Given the asphaltene content, the results indicate distinct oxidation and compositional differences in the continuous phase, likely attributed to the influence of unreproducible atmospheric factors such as water and ultraviolet radiation.

As it is possible to observe from previous results, the aging causes the stiffening of bitumen with a reduced molecular flow, which is verified by the increase in viscosity. The laboratory aging procedure seems to be only in agreement with natural aging because of the similar asphaltene quantity, rather than with the quantity of the aromatic fraction of bitumen, which transforms into the resins and after into asphaltenes as obtained by the *n* parameter. 

### 3.2. Rheology of Modified Samples 

**Figure 4:**
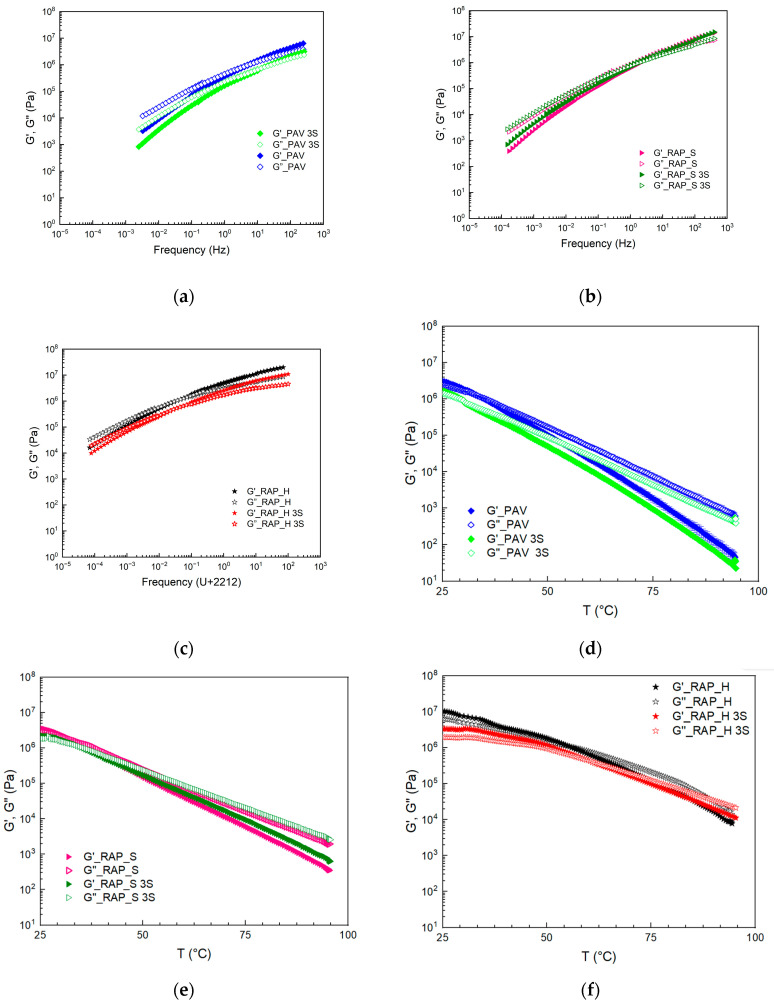
G′ and G″ results in TTS plot (**a**–**c**) and in Time Cure plots (**d**–**f**) for PAV (**a**,**d**), RAP_S (**b**,**e**) and RAP_H (**c**,**f**).

SO was used to flux both laboratory-aged bitumen and RAPbs, and the results of the rheological analysis are reported in Figure 4a–f, where the TTS and Time Cure results are reported in terms of G′ and G″. 

The results of all samples analyzed are compared with the starting bitumen. The addition of soybean oil to laboratory-aged bitumen, PAV, leads to a softening of the matrix. Both moduli, G′ and G″, decrease, and, with the addition of the oil, both have an increase in the crossover frequency and an increase in the slope of the curves, which is also possible to see from the Winter model parameter *n*. Both of these effects can be attributed to a weakening effect of the oil in accordance with what is expected in the literature [17]. 

The comparison of G′ and G′ trends with temperature (Figure 4d) also confirms the weakening effect of the addition of SO. In particular, both moduli decrease and slightly change the crossover temperature at which the material behavior changes from solid to liquid-like. 

The addition of SO was also carried out on RAPbs, hard and soft samples, and rheological characterization was performed. Looking carefully at the TTS results on the soft RAP samples, it can be seen that the G′ and G′ moduli are very similar when comparing the binder with and without SO. The addition of the oil does not seem to lead to any major changes in the mechanical behavior of the binder. Contrary to expectation, there is a slight increase in moduli with the oil addition at a low frequency (in the range of 0.001–0.1 Hz), while the crossover frequency, f_c_, remains unchanged. The same effect is confirmed in the Time Cure test, in which slight differences in the moduli G′ and G″ are observed, with slightly higher values for the sample with the addition of oil. In particular, it can be observed that the moduli are lower at low temperatures, before the crossover, due to the addition of the SO, while the moduli begin higher with temperature. As reported in the literature, this effect can be due to the interaction between the polar molecules of RAP_S with the triglycerides of SO, leading to the formation of a more consistent system [29]. Probably, however, this effect is evident at precise ratios between polar species in the bitumen and triglycerides. In fact, looking at the samples obtained with SO and RAP_H, it can be noted that the moduli of the modified samples are lower in the analyzed range. In both cases, however, it is observable that the SO lowers the modulus at low temperatures, acting like waxes, while it somehow seems to have fewer benefits at medium and high temperatures for both RAPbs.

Comparing the natural with the laboratory-aged bitumen, it can also be speculated that the SO acts on the structure of the PAV and RAP_S in the same way due to the similar asphaltene quantity. They have, after modification, similar *n* values (Table 2) due to the dilution action on the continuous phase but different consistency attributable to the value of *k*. The trend is also confirmed by viscosity data.

In fact, viscosity measurements were also carried out for the SO samples as a key parameter to control the regeneration of bitumens and their recovery to initial application conditions. According to the oscillatory tests, the addition of SO to the PAV bitumen at both 60 °C and 75 °C led to an evident decrease in viscosity values over the entire shear rate range examined, making the aged bitumen more workable and less viscous. The reduction in viscosity shows how SO is suitable to act as a fluxing agent if we look at the laboratory-aged bitumen. According to the literature, the presence of SO at higher controlled concentrations can also result in a decrease in viscosity and improved processability of the material [20]. As reported by other authors, the softening of the matrix is caused by the polar carboxyl group of the fatty acids in the oil [27]. The carboxylic group interferes with the weak bonds existing between the polar molecules of the asphalt binder decreasing the interactions and, as a consequence, the bitumen viscosity. In addition to this, there is also a size-reducing effect of the more polar molecular agglomerates within the asphalt binder that leads to a higher fluidity of the bitumen [27]. The same effect is not evident for RAPbs.

**Figure 5:**
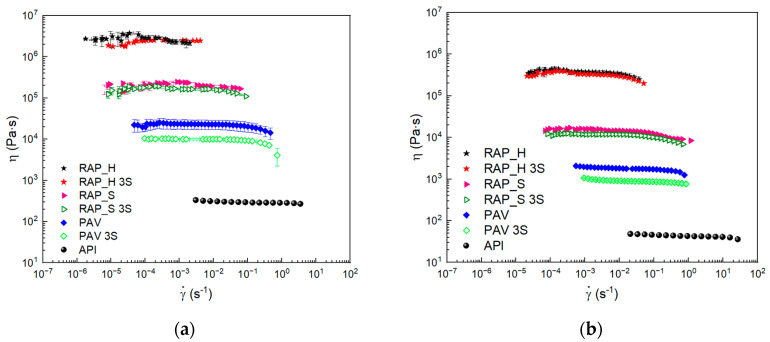
Flow curve plot: (**a**) at 60 °C; (**b**) at 75 °C for samples before and after SO addition.

In Figure 5a,b, the viscosity measurements on RAP_H and RAP_S were reported. It can be seen from the graphs how the addition of SO to the two different RAP bitumens acts differently. It is evident that RAP_S and RAP_H with SO addition at 3% *w*/*w* do not change their viscosity compared to the sample without oil. This effect can be due to a stronger physical interaction of the polar molecules of RAP_S and H with the triglycerides of SO, leading to the formation of a more viscous network at the SO level added [29]. The different effects of SO on viscosity can be due to the different actions of the polar carboxyl group of the fatty acids present in the vegetable oil. They can weaken differently the secondary bonds between polar asphalt bitumen molecules because of the different dispersed phase compositions of the two aged bitumens. It is possible, as reported by the literature, that, for RAP_S, a higher amount of SO is required to obtain the fluxing effect, as well as for RAP_H [27]. The not-flux effect can be imputable to a higher quantity of large-size molecules in the aged binders, according to [27]. 

## 4. Conclusions

The study of aging in road asphalts is fundamentally important, both for understanding how pavements degrade and for exploring the possibility of recycling worn asphalt pavements. Recycling often requires adding fluxing or rejuvenator agents to restore the workability needed for paving processes. Therefore, studies on their effects on aged binders are necessary. 

In light of the above, this work investigated the effect of aging on the rheological properties of 70/100 virgin bitumen using the PAV aging technique. The results were compared with two binders extracted from RAP (reclaimed asphalt pavement): a soft bitumen obtained from virgin aggregates and a hard asphalt obtained from RAP aggregates. From the rheological investigation, useful information has been provided that can be taken into account in studying and preparing mixes with aged bitumens. 

The laboratory PAV aging technique cannot perfectly predict natural aging. Although the asphaltene content in the laboratory-aged sample reached a level similar to one of the naturally aged binders, the G′ and G″ moduli are still higher in the naturally aged material. 

However, the transition temperature from liquid to solid behavior and the frequency dependence are similar for aged bitumen with similar asphaltene content. Notably, the gel strength and viscosity values at all temperatures are higher for the RAP binders (RAP_S and RAP_H) compared to the PAV sample.

The addition of a 3% *w*/*w* of SO was also investigated to evaluate its effect on lowering the viscosity of RAP binders. 

The SO addition softened the PAV sample, indicated by a decrease in both mechanical moduli and dynamic viscosity. 

No significant change in viscosity was observed for RAP_S and RAP_H. This suggests that the structuring and/or weakening effect of SO is highly dependent on the chemical and physical characteristics of the starting bitumen and cannot be universally applied. 

Finally, this study highlights the need for further research on bitumen aging and the impact of additives, with the ultimate goal of improving pavement recycling techniques and maximizing the reuse of asphalt materials.

## Figures and Tables

**Table 1:** SARA composition of virgin bitumen.

SARA Component	% *w*/*w*
Satures	5.70
Aromatics	48.60
Resins	20.90
Asphaltenes	25.80

**Table 2:** Data of crossover frequency (f_c_), crossover temperature (T_c_) and Winter model parameters, *k* and *n*, with regression index R^2^.

ID Sample	f_c_, Hz	T_c_, °C	*k*, Pas^n^	*n*, -	R^2^
API	-	-	50,100 ± 900	0.718 ± 0.004	0.999
PAV	6.5 ± 2.0	33.5 ± 1.0	691,000 ± 15,000	0.438 ± 0.005	0.996
RAP_S	4.5 ± 0.5	32.4 ± 0.5	1,390,000 ± 30,000	0.417 ± 0.005	0.995
RAP_H	0.042 ± 0.011	58.0 ± 0.4	5,728,000 ± 60,000	0.329 ± 0.004	0.995
PAV 3S	14.7 ± 2.4	30.0 ± 0.5	316,000 ± 6000	0.462 ± 0.004	0.998
RAP_S 3S	2.05 ± 0.66	34.5 ± 0.3	1,101,000 ± 10,000	0.459 ± 0.002	0.999
RAP_H 3S	0.045 ± 0.020	59.5 ± 1.0	3,098,000 ± 50,000	0.308 ± 0.005	0.988

## Data Availability

The original contributions presented in the study are included in the article, further inquiries can be directed to the corresponding author.
